# Zhizhu decoction alleviates slow transit constipation by regulating aryl hydrocarbon receptor through gut microbiota

**DOI:** 10.1080/13880209.2022.2157020

**Published:** 2022-12-23

**Authors:** Yong Wen, Yu Zhan, Shiyu Tang, Fang Liu, Rong Wu, Pengfei Kong, Qian Li, Xuegui Tang

**Affiliations:** a Hospital of Chengdu University of Traditional Chinese Medicine, Chengdu, China; b Department of Traditional Chinese Medicine, The Affiliated Hospital of Southwest Medical University, Luzhou, China; c Affiliated Hospital of Integrated Chinese Medicine and Western Medicine, Chengdu University of Traditional Chinese Medicine, Chengdu, China; d Department of Anus and Intestine Surgery, Chengdu Integrated TCM & Western Medicine Hospital, Chengdu, China; e Department of Integrated Traditional and Western Medicine Anorectal, Affiliated Hospital of North Sichuan Medical College, Nanchong, China; f Chengdu University of Traditional Chinese Medicine, Chengdu, China

**Keywords:** Intestinal neurotransmitters, intestinal motility, metabolomics, 16S rRNA sequencing, functional constipation

## Abstract

**Context:**

Slow transit constipation (STC), the most common type of constipation, seriously affects the life of patients. Zhizhu decoction (ZZD), a traditional Chinese medicine compound, has is effective against functional constipation, but the mechanism is still unclear.

**Objective:**

This research explores the mechanism of ZZD on STC from the perspective of metabolomics and gut microbiota.

**Materials and methods:**

Fifty-four C57BL/6 mice were randomly divided into six groups (*n* = 9): control (control); STC (model); positive control (positive); low-dose (5 g/kg; L-ZZD), medium-dose (10 g/kg; M-ZZD), and high-dose (20 g/kg; H-ZZD) ZZD treatment. Following treatment of mice with ZZD for two weeks, the changes in intestinal motility, colon histology, intestinal neurotransmitters, and aryl hydrocarbon receptor (AHR) pathway determined the effects of ZZD on the pathophysiology of STC. LC-MS targeting serum metabolomics was used to analyze the regulation of ZZD on neurotransmitters, and 16S rRNA high-throughput sequencing was used to detect the regulation of the gut microbiome.

**Results:**

ZZD had the highest content of naringin (6348.1 mg/L), and could significantly increase the 24 h defecations (1.10- to 1.42-fold), fecal moisture (1.14-fold) and intestinal transport rate (1.28-fold) of STC mice, increased the thickness of the mucosal and muscular tissue (1.18- to 2.16-fold) and regulated the neurotransmitters in the colon of STC mice. Moreover, ZZD significantly activated the AHR signaling pathway, and also affected the composition of gut microbiota in STC mice.

**Discussion and conclusions:**

The beneficial effect and the possible mechanism of ZZD on STC could provide a theoretical basis for the broader clinical application of ZZD.

## Introduction

Slow transit constipation (STC) is a type of constipation caused by the obstruction of colon motility, resulting in the retention of colon content and slow transportation of the colon, which seriously affects the quality of life of patients (Wang 2015). The main purpose of STC treatment is to relieve constipation symptoms and restore normal intestinal movement and defecation function (Black and Ford 2018). Western medicine or surgical treatment is mainly used, but these treatment methods often have serious adverse reactions or postoperative complications, which further increase the pain of patients (Sharma and Rao 2017). The etiology of STC is complex, involving not only a variety of hormones and neurotransmitters but also a variety of gastrointestinal microstructure abnormalities, leading to difficulties in drug treatment (Camilleri et al. 2017). Safe and effective drugs for regulating intestinal peristalsis and effective treatment of STC are still clinical needs.

Traditional Chinese medicine has a long history of use for digestive system diseases and has shown unique effects in the treatment of constipation (Lin et al. 2009; Wang et al. 2012; Li et al. 2017). Zhizhu decoction (ZZD), composed of Zhishi (*Citrus* *×* *aurantium* L.) and Baizhu (*Atractylodes macrocephala* Koidz), has been used to treat functional dyspepsia and gastric ulcer. Clinical studies have shown that ZZD has a good therapeutic effect on functional dyspepsia of spleen-deficiency and qi-stagnation syndrome (Wu et al. 2011). In addition, ZZD can promote gastric emptying and protect the gastric mucosa from ethanol-induced ulcers in rats (He et al. 2013). The beneficial effects of ZZD on functional constipation and gastrointestinal tract suggest that it may also have beneficial effects on STC, but there is still a lack of systematic research on the therapeutic effects of ZZD on STC, and the underlying mechanism of ZZD regulating intestinal peristalsis to improve constipation is still unclear. It is necessary and urgent to study the therapeutic effect and mechanism of ZZD on STC, which can provide new ideas for the treatment of STC.

Numerous studies have shown that chronic constipation leads to an imbalance of gut microbiota, with a relative decrease in certain bacteria and a relative increase in potentially disease-causing microbes (Ohkusa et al. 2019). The gut microbiome has become a key regulator of intestinal tissue physiology (Shi et al. 2017). The disruption of the gut microbiome interferes with endogenous metabolites (Hyland and Cryan 2016), resulting in reduced excitability of intestinal neurons, altered motor procedures, and prolonged intestinal transfer time (Yoo and Mazmanian 2017). In addition, a recent study has shown that transcription factor aryl hydrocarbon receptor (AHR), as a biosensor of the intestinal neural network, can sense the microbial environment in the intestinal cavity, participate in maintaining the excitability of intestinal neurons and regulate the intestinal physiological functions (Obata et al. 2020). The gut microbiome regulating AHR signaling pathway is closely related to STC, which may be a potential target for STC treatment.

Based on this, we speculated that the therapeutic effects of ZZD on STC might be related to the metabolites of the host and intestinal microorganisms. To explore the potential mechanism of ZZD on STC, an STC mice model was established by compound diphenoxylate and ZZD was used to regulate intestinal function. The effects of ZZD on the pathophysiology of STC were investigated by assessing the intestinal motility and histology of the colon. The possible mechanism was explored by detecting the changes of neurotransmitters and AHR signaling pathway in the colon of mice. In addition, LC-MS targeting serum metabolomics was used to analyze the changes of neurotransmitters in the serum of mice, and 16S rRNA gene high-throughput sequencing was used to monitor the structure and composition of the gut microbiome to determine the changes in species abundance caused by ZZD. This study elucidated the therapeutic mechanism of STC and provided a new approach and theoretical support for the clinical application of ZZD.

## Materials and methods

### Preparation of ZZD

ZZD is a classic prescription, which has been clinically proven to have high safety. It was prepared with the assistance of the Traditional Chinese medicine pharmacy of Chengdu First People’s Hospital, and the method was consistent with our previous study (Wen et al. 2022). Zhishi and raw Baizhu, the raw materials for the preparation of ZZD, were obtained from the Traditional Chinese medicine pharmacy of Chengdu First People’s Hospital in 2020, and verified by the Chinese pharmacists in our hospital according to the Chinese Pharmacopoeia, and the medicinal materials were stored in Chengdu First People’s Hospital. Water was added to a mixture of 15 g Zhishi and 60 g raw Baizhu at a ratio of 10:1 (water:plant material, v/w). After 30 min of soaking in water, the plant material was boiled for 30 min and filtered. Then, the dregs were re-boiled for 30 min after the addition of fresh water at a ratio of 8:1 (water:plant material, v/w). Finally, the dregs were filtered, and the filtrates were combined and concentrated into a 1 g/mL decoction. The ZZD was stored at 4 °C before use.

### High-performance liquid chromatography analysis

The active components of ZZD were analyzed by Shimadzu LC AT-20 HPLC with double solvent pump high-pressure gradient system and SPD-20A photodiode array detector (Shimadzu, Kyoto, Japan). The chromatography was performed on 5 μm particles, 250 × 4.6 mm, C_18_ column (35 °C) at a flow rate of 1.0 mL/min and eluted with 0.1% formic acid aqueous solution (A) and 0.1% formic acid acetonitrile (B), and the gradient elution was 0–7 min, 23% B; 7–17 min, 23–80% B; 17–20 min, 80–100% B; 20–30 min, 100% B. Naringin (CAS No.: 10236-47-2), Hesperetin (CAS No.: 520-33-2), Atractylenolide-III (CAS No.: 73030-71-4), Atractylenolide-II (CAS No.: 73069-14-4) and Atractylenolide-I (CAS No.: 73069-13-3) were purchased from Solarbio Life Sciences Co., Ltd. (China) as the standard products for the preparation of standard curves. The retention times (RT) of the peaks were compared with those measured in the standard samples to identify the compounds, and the content of each compound was obtained by the standard curve method.

### Animal treatment

A total of fifty-four 6-week-old SPF C57BL/6 male mice, weighing 20–25 g, were obtained from Chengdu Dossy experimental animals [China, License number: SCXK (Chuan) 2020-030] and housed in an SPF experimental animal center. This study was carried out in strict accordance with the Guide for the Care and Use of Laboratory Animals of the National Institutes of Health. All experimental protocols of the present study were approved by the Animal Ethics Committee of North Sichuan Medical College (NSMC Ethical Animal Audit [2021] No. 82; Nanchong, China), and all efforts were made to minimize animal suffering and to reduce the number of animals used. Following a week of adaptation, mice were randomly divided into six groups (*n* = 9): Control (control), STC model (model), positive control (positive), low-dose ZZD treatment (L-ZZD), medium-dose ZZD treatment (M-ZZD) and high-dose ZZD treatment (H-ZZD). The mice were given 40 mg/kg Compound Diphenoxylate Tablets (CD, Changzhou Kangpu Pharmaceutical, China) by gavage at 19:00 every night to establish the STC model for 2 consecutive weeks. The positive control group was intragastrically given 3 mg/kg mosapride daily, and the L-ZZD, M-ZZD, and H-ZZD group were given 10, 15, and 20 g/kg ZZD, respectively, based on the human-animal drug dose conversion table. The medium dose of ZZD was approximately the clinical dose of 75 g. All treatment groups were treated for 3 weeks from the second week of gavage CD. Mice in the control group received an equivalent volume of saline. All mice were allowed free access to water and food. Body weight and 24 h defecations were recorded weekly.

### Sample collection

On the last day of the treatment, three pellets of fresh fecal samples from each mouse were collected, weighed, and placed in a 60 °C oven for 12 h, then weighed again to calculate the fecal moisture. And after 4 weeks of treatment, three mice of each group were randomly selected to fast for 18 h and given 0.5 mL semi-solid paste containing 5% black activated carbon. After 30 min, the mice were killed by cervical dislocation, the entire intestine from the pylorus to the end of the colon was collected and the distance from the pylorus to the front of the black semi-solid paste and the end of the colon was measured to calculate the intestinal transport rate (ITR). ITR (%) = distance from the pylorus to the leading edge of black semisolid paste (cm)/distance from the pylorus to the end of the colon (cm) × 100. In addition, the other six mice in each group were sacrificed, blood samples were collected and centrifuged at 3000 rpm for 15 min at 4 °C to obtain serum for serum metabolomics analysis, and the fresh feces in the colon were collected aseptically and rapidly frozen in liquid nitrogen for 16S rRNA sequencing. Moreover, all the colon samples of nine mice were collected, part of which were stored at −80 °C for molecular detection, and the others were fixed with 4% paraformaldehyde for pathological detection.

### Hematoxylin-eosin staining

The colonic tissues were fixed in 4% paraformaldehyde, embedded in paraffin wax, and then cut into 3 μm sections. The sections were first dewaxed with xylene and ethanol gradient and then stained with hematoxylin and eosin staining for pathological studies. Each section of colonic tissue was evaluated by a light microscopy (Nikon, Japan).

### Enzyme-linked immunosorbent assay

The expressions of acetylcholine (Ach), substance P (SP), 5-hydroxytryptamine (5-HT), and vasoactive intestinal peptide (VIP) in the colon were detected by ELISA. First, the colon tissue was thoroughly ground with PBS on the ice. The homogenate was centrifuged at 10,000 rpm for 10 min and the supernatant was taken. The protein content was determined by the BCA protein quantification kit (Solarbio, China), and the consistency of the concentration was adjusted for ELISA. The ELISA operation was carried out according to the kit instructions (ZCIBIO Technology Co., Ltd, China) with the double antibody sandwich enzyme-linked immunosorbent method. In brief, the specific anti-mice Ach, SP, 5-HT, and VIP antibodies were precoated on a high-affinity enzyme-labeling plate to form a solid phase antibody, then bound to the corresponding antigen in the tissues to form an immune complex. After washing, enzyme-labeled antibodies were added to combine with the antigen in the immune complex to form the enzyme-labeled antibody-antigen-solid antibody complex. Finally, the substrate was added for color development, and the depth of color was proportional to the concentration of the sample, to determine the content in the sample.

### Western blot analysis

The protein expressions of AHR and Cytochrome P450, Family 1, Subfamily A, Polypeptide 1 (CYP1A1) in colonic tissues were detected by WB assay. The colonic tissue was homogenized in radioimmunoprecipitation assay buffer (RIPA) containing protease and phosphatase inhibitors (Solarbio, China) and lysed on ice for 30 min. The lysate was centrifuged at 10,000 rpm at 4 °C for 10 min to collect the supernatant. Total protein concentration was detected using the BCA protein quantification kit (Solarbio, China). Subsequently, the protein sample was separated by 10% sodium dodecyl sulfate-polyacrylamide gel electrophoresis (SDS-PAGE) and then transferred into polyvinylidene fluoride (PVDF) membranes (Sigma-Aldrich, USA). The primary anti-AHR (1:1000, cat. No. sc-133088) and anti-CYP1A1 (1:1000, cat. No. sc-393979) antibodies (Santa Cruz Biotechnology, USA) were used to incubated overnight at 4 °C. Following washing, the PVDF membranes were incubated with HRP-conjugated secondary antibody (1:4000, cat. No. sc-2005; Santa Cruz Biotechnology, USA) for 1 h at room temperature. The expression of the relative protein in colon tissue was visualized with an enhanced chemiluminescence detection kit (Bio-Rad Laboratories, Inc.) using Tanon 5200 (Tanon, Shanghai, China) and quantified by the Image Lab analysis software version 4.1 (Bio-Rad, California, USA).

### Real-time quantitative polymerase chain reaction

RNA extraction and RT-PCR analysis were performed according to kit instructions. Briefly, the total RNA of colonic tissue was prepared with an RNA Sample Total RNA kit (TIANGEN, Beijing, China) following the manufacturer’s instructions. The cDNA was obtained using a FastKing RT kit (With gDNase) (TIANGEN, Beijing, China) by incubating at 42 °C for 15 min and then at 95 °C for 3 min. Quantitative real-time PCR was run using a ChamQ™ SYBR qPCR Master Mix (Vazyme biotech, Nanjing, China) with a CFX96™ RT-PCR detection system (Bio-Rad, Singapore) at a final volume of 20 µL using the standard protocol, which was as follows: 95 °C for 30 s, 40 cycles of 95 °C for 10 s and 60 °C for 30 s, 95 °C for 15 s, 60 °C for 60 s, and 95 °C for 15 s. Relative gene expression levels of *Ahr* and *Cyp1a1* were determined using the comparative 2^-ΔΔCT^ method using *Actin* as endogenous control. The primer sequences in this study were shown in Table 1.

**Table 1.** The primer sequences were used for Real-Time PCR assay in mice.

Genes	Forward primer	Reverse primer
*Ahr*	5′-CACAGAGTTAGACCGCCTGG-3′	5′-GGGGTGGACTTTAATGCAACATC-3′
*Cyp1a1*	5′-AAGGGCATAGGCAGCCAC-3′	5′-TCTTCAGGCCTTTGGGAACC-3′
*Actin*	5′-GAAGATCAAGATCATTGCTCC-3′	5′-TACTCCTGCTTGCTGATCCA-3′

### Immunofluorescent staining

Under an anatomical microscope, the mucosa and submucosa of colonic tissues were removed as a layer from the colonic tissues fixed at 4% paraformaldehyde to prepare full-thickness tissue specimens of colonic musculus, and the full-thickness tissue of the colonic musculus was used for immunofluorescence staining. The specimens were antigen repaired and sealed by bovine serum albumin for 1 h. Then the primary anti-AHR (1:200, cat. No. sc-133088) and anti-CYP1A1 (1:200, cat. No. sc-393979) antibodies (Santa Cruz Biotechnology, USA) were incubated with anti-PGP9.5 antibody (1:200, cat. No. ab10404, Abcam, UK) at 4 °C overnight, and the fluorescent secondary antibodies Alexa Fluor 647-labeled Goat Anti-Rabbit IgG(H + L) (1:500; cat. No. A0468; Beyotime Biotechnology) and Alexa Fluor 488-labeled Goat Anti-Mouse IgG (H + L) (1:500; cat. No A0428; Beyotime Biotechnology) were used to incubating at room temperature and away from light for 1 h. Finally, the 4′,6-diamidino-2-phenylindole (DAPI, Sigma, USA) was used for re-staining the nucleus for 5 min, and images were scanned and obtained under OlyVIA (OLYMPUS, Japan). The Integrated Density (IntDen) and Area of each image were determined by image-J analysis system (National Institutes of Health, USA), and the mean fluorescence intensity of each image was calculated.

### Serum metabolomics analysis

The mouse serum was added with 400 μL 10% formic acid methanol solution-ddH_2_O (1:1), centrifuge at 12,000 rpm at 4 °C for 5 min, and the supernatant was collected. The 100 μL supernatant or 100 μL diluted 100 times supernatant was mixed with 100 μL 100 ppb double isotope internal standard, then filtered through a 0.22 μm membrane. The composition and content of the neurotransmitter in the serum of mice were analyzed by LC-MS targeting metabolomics using Waters ACQUITY UPLC and AB triple quadrupole Mass Spectrometer (USA). The 23 kinds of neurotransmitter standards were prepared in single standard mother liquor with 10% formic acid methanol. Each mother liquor was measured to make a mixed standard, which was diluted one by one with 10% formic acid methanol to a suitable concentration to make a working standard solution. The information on neurotransmitters and their concentration were shown in Table 2. The chromatography was performed on an ACQUITY UPLC BEH C18 column (2.1 × 100 mm, 1.7 μm, Waters, USA) and eluted with 10% methanol aqueous solution (containing 0.1% formic acid; A) and 50% methanol aqueous solution (containing 0.1% formic acid; B) (0–1 min 20–100% B; 1–7 min, 100% B; 7–7.5 min 100–20% B; 7.5–11 min 20% B) at a flow rate of 0.4 mL/min. The mass spectrometric was performed using an electrospray ionization (ESI) source with positive ion ionization mode. The temperature of the ion source is 500 °C, the voltage of the ion source is 5500 V, the collision gas is 6 psi, the curtain gas is 30 psi, and the atomizing gas and the auxiliary gas are 50 psi. The scan was performed using multiple response monitoring (MRM). Based on the detection results, targeted quantitative analysis was carried out on the detected samples, and relevant data were analyzed according to the quantitative results.

**Table 2.** Standard curve concentrations of neurotransmitters.

Neurotransmitters	Standard curve concentrations (ng/mL)											
	1	2	3	4	5	6	7	8	9	10	11	12
GABA (4-Aminobutyric acid, CAS: 56-12-2)	1000	500	250	200	100	50	40	20	10	5	2	1
HisA (Histamine, CAS: 51-45-6)	–	250	125	100	50	25	20	10	5	2.5	1	0.5
PA (Picolinic acid, CAS: 98-98-6)	2500	1250	625	500	250	125	100	50	25	12.5	5	–
TyrA (Tyramine, CAS: 51-67-2)	500	250	125	100	50	25	20	10	5	2.5	1	–
Ach (Acetylcholine chloride, CAS: 60-31-1)	200	100	50	40	20	10	8	4	2	1	0.4	–
Gln (L-Glutamine, CAS: 56-85-9)	1000	500	250	200	100	50	40	20	10	5	2	1
Glu (L-Glutamic acid, CAS: 56-86-0)	2000	1000	500	400	200	100	80	40	20	10	4	–
DA (Hydroxytyramine hydrochloride, CAS: 62-31-7)	500	250	125	100	50	25	20	10	5	2.5	1	0.5
His (L-Histidine, CAS: 71-00-1)	2000	1000	500	400	200	100	80	40	20	10	4	2
TrpA (Tryptamine, CAS: 61-54-1)	–	100	50	40	20	10	8	4	2	1	0.4	0.2
NE (Noradrenaline hydrochloride, CAS: 55-27-6)	1000	500	250	200	100	50	40	20	10	5	–	–
5-HT (Serotonin hydrochloride, CAS: 153-98-0)	1000	500	250	200	100	50	40	20	10	5	–	–
Tyr (L-Tyrosine, CAS: 60-18-4)	2500	1250	625	500	250	125	100	50	25	12.5	5	2.5
E (Adrenaline hydrochloride, CAS: 329-63-5)	250	125	62.5	50	25	12.5	10	5	2.5	–	–	–
KynA (Kynurenic acid, CAS: 492-27-3)	1000	500	250	200	100	50	40	20	10	5	–	–
5-HIAA (5-Hydroxyindole-3-acetic acid, CAS: 54-16-0)	1000	500	250	200	100	50	–	–	–	–	–	–
DOPA (Levodopa, CAS: 59-92-7)	2500	1250	625	500	250	125	100	50	25	12.5	5	–
Trp (L-Tryptophan, CAS: 73-22-3)	500	250	125	100	50	25	20	10	5	2.5	1	0.5
XA (Xanthurenic acid, CAS: 59-00-7)	2000	1000	500	400	200	100	80	40	20	–	–	–
Kyn (DL-Kynurenine, CAS: 343-65-7)	2000	1000	500	400	200	100	80	40	20	10	4	–
VMA (Vanillymandelic Acid, CAS: 55-10-7)	5000	2500	1250	1000	500	250	200	100	50	–	–	–
5-HTP (5-Hydroxytryptophan, CAS: 4350-09-8)	500	250	125	100	50	25	20	10	5	2.5	–	–
MT (Melatonine, CAS: 73-31-4)	100	50	25	20	10	5	4	2	1	0.5	–	–

### DNA extraction and 16S rRNA gene sequencing

Intestinal contents of the mice were collected when mice were sacrificed, and total bacterial DNA was extracted from the fecal samples using a TIANamp Stool DNA Kit (TIANGEN, Beijing, China) following the manufacturer’s instructions. The DNA integrity and concentration were measured by agarose gel electrophoresis and a nanodrop instrument (Thermo, USA), respectively. Illumina MiSeq platform was used for paired-end sequencing of bacterial community DNA fragments.

### Statistical analysis

All data were analyzed using SPSS 22.0 (IBM, USA) statistical analysis software, and shown as mean ± standard deviation (*SD*). A two-tailed Student’s *t*-test, one-way or two-way analysis of variance (ANOVA) was applied to analyze the significant differences between groups, *p* < 0.05 was considered as a statistically significant difference.

## Results

### The components of ZZD were analyzed by HPLC

Zhishi contains a variety of flavonoids, which have the function of regulating gastrointestinal functions (Lee et al. 2009). Atractylenolide-III, atractylenolide-II, and atractylenolide-I are the main effective components of Baizhu to regulate and protect the gastrointestinal system (Wang et al. 2010). The contents of naringin, hesperetin, atractylenolide-III, atractylenolide-II, and atractylenolide-I in ZZD were detected by HPLC. As shown in Table 3, the contents of five compounds were obtained by standard curve. Among them, naringin had the highest content (6348.1 mg/L), followed by hesperetin (11.7 mg/L), atractylenolide-III (8.7 mg/L), atractylenolide-II (0.7 mg/L), and atractylenolide-I (0.08 mg/L).

**Table 3.** HPLC analysis results of ZZD.

Compound name	Molecular formula	RT (min)	Amount (mg/L)
Naringin	C_27_H_32_O_14_	16.06	6348.1
Hesperetin	C_16_H_14_O_6_	26.817	11.7
Atractylenolide-III	C_15_H_20_O_3_	33.793	8.7
Atractylenolide-II	C_15_H_20_O_2_	37.532	0.7
Atractylenolide-I	C_15_H_18_O_2_	40.02	0.08

### Effects of ZZD on intestinal motility in STC mice

**Figure 1.**
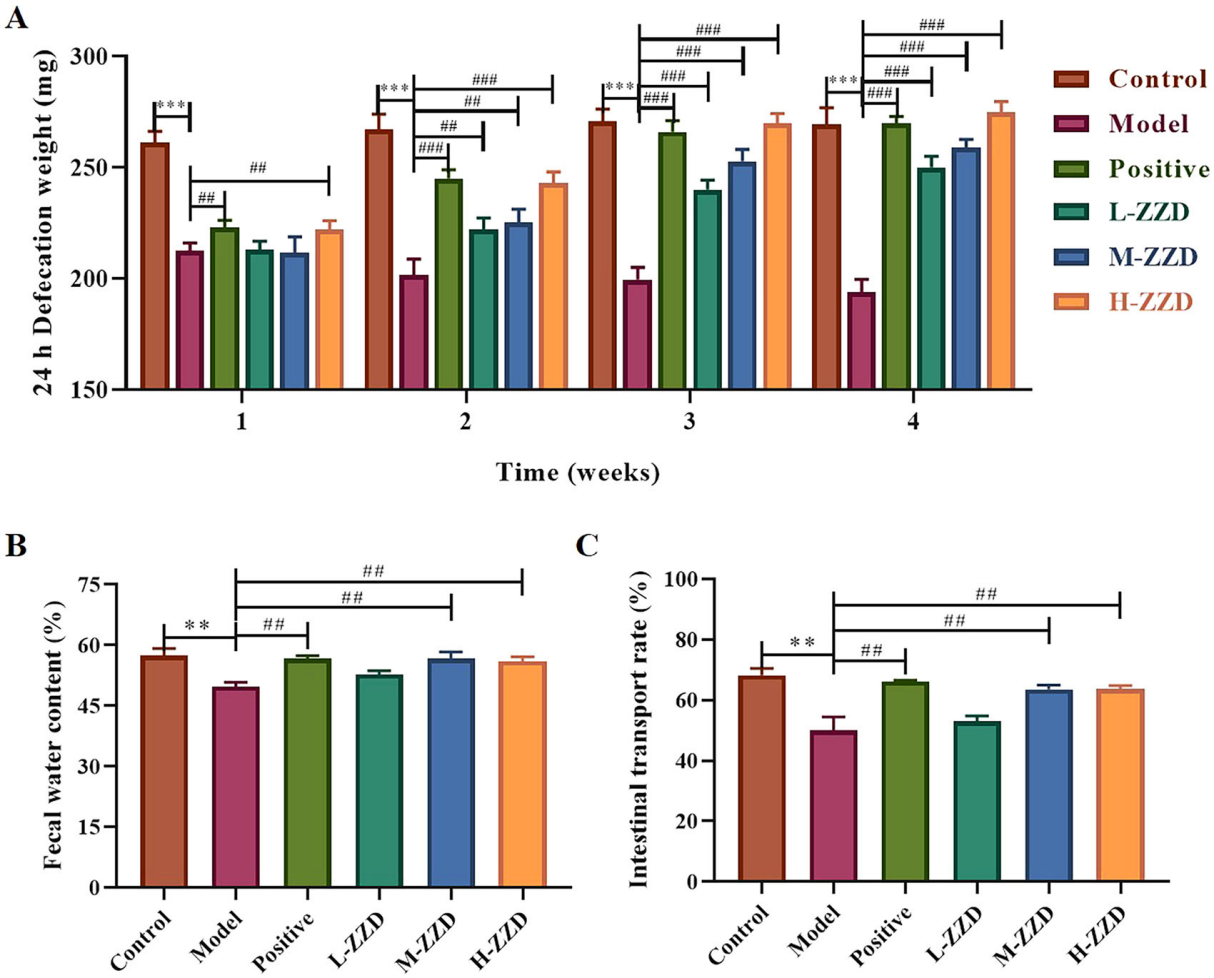
Effects of ZZD on intestinal motility of STC mice. (A) 24 h defecation weight of STC mice at the various indicated time points. (B) The fecal water content of mice. (C) The intestinal transport rate of mice. Data were shown as mean ± *SD*, *n* = 9. **p < 0.01, ***p < 0.001, compared with the control group; ^##^*p* < 0.01, ^###^*p* < 0.001, compared with the STC model group. ZZD: Zhizhu Decoction; L-ZZD: low-dose ZZD treatment group; M-ZZD: medium-dose ZZD treatment group; H-ZZD: high-dose ZZD treatment group.

The intestinal motility of mice in different groups was evaluated by 24 h defecation weight, fecal moisture, and ITR, and it was found that compared with the control group, the 24 h defecation weight, fecal moisture, and ITR of mice in the STC model group was significantly reduced, which was significantly reversed after all doses of ZZD treatment (*p* < 0.05, Figure 1). These results indicate that ZZD can restore intestinal motility in STC mice and exert therapeutic effects on gastrointestinal function.

### Effects of ZZD on colonic tissue morphology in STC mice

**Figure 2.**
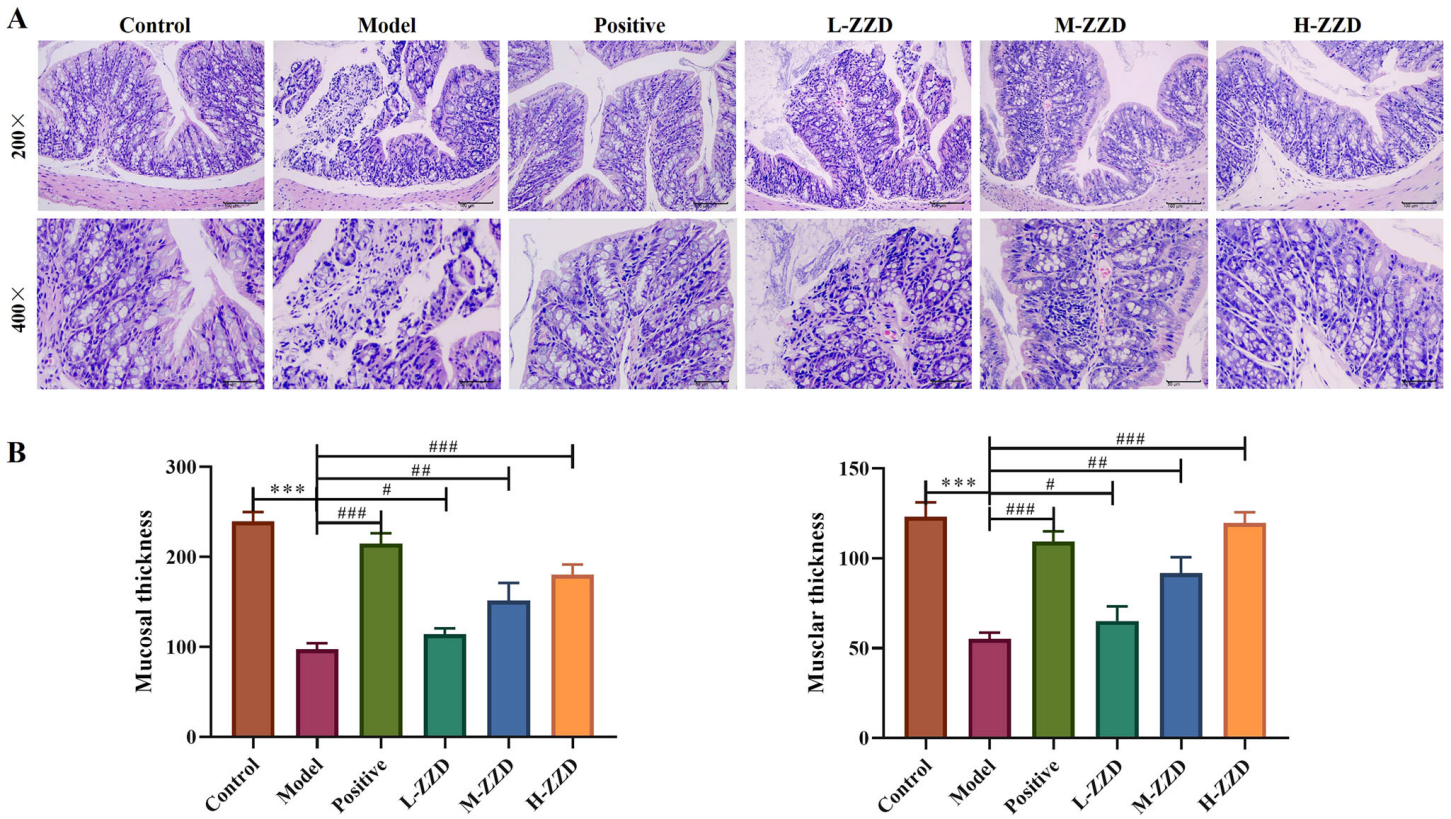
Effects of ZZD on histological changes in STC model mice. (A) Representative images showing hematoxylin and eosin of colon tissues in mice (magnification, ×200 and/or ×400). (B) Tissue thickness of mucosal and muscular layers. Data were shown as mean ± *SD*, *n* = 9. ***p < 0.001, compared with the control group; ^#^*p* < 0.05, ^##^*p* < 0.01, ^###^*p* < 0.001, compared with the STC model group. ZZD: Zhizhu Decoction; L-ZZD: low-dose ZZD treatment group; M-ZZD: medium-dose ZZD treatment group; H-ZZD: high-dose ZZD treatment group.

Histopathological analysis of the colonic tissue showed that the colonic tissue of mice in the control group had complete structures of mucosa, submucosa, muscularis and outer membrane, the mucosa was covered with single columnar epithelial cells, and a large number of intestinal glands were found in the lamina propria. However, in the model group, some areas of colonic tissue were characterized by mucosal necrosis, loss of epithelial cells in the necrotic area, necrotic of intestinal glands in the lamella propria, and the tubular structure was absent, only necrotic cell fragments and a few lymphocytes or neutrophils were observed. After treatment with all doses of ZZD and positive drugs, the damage to colonic tissue was significantly improved. The colon structure was relatively complete, the epithelial cells were arranged in a more orderly manner, and only a few intestinal glands were necrotic in the lamina propria, with slight inflammatory cell infiltration (Figure 2(A)). In addition, the thickness of mucosa tissue and muscle layers was also determined. The analysis revealed that compared with the control group, the tissue thickness was significantly reduced in the model group. However, the above effects were significantly reversed following mice treatment with different doses of ZZD (*p* < 0.05; Figure 2(B)).

### Effects of ZZD on intestinal neurotransmitters in STC mice

**Figure 3.**
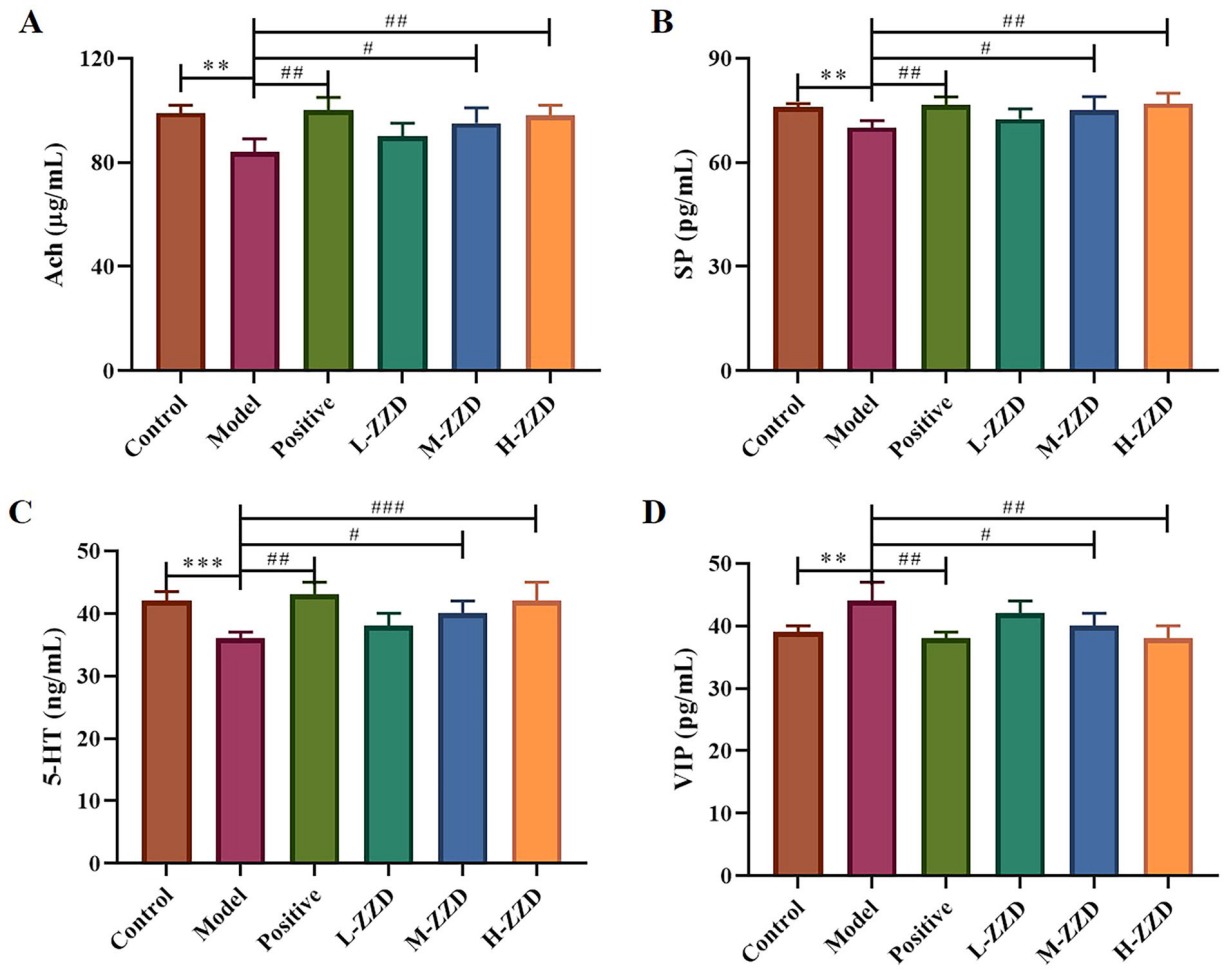
Effects of ZZD on neurotransmitters in the colon of STC mice. (A) The relative content of Ach in the colon of STC mice. (B) The relative content of SP. (C) The relative content of 5-HT. (D) The relative content of VIP. Data were shown as mean ±* SD*, *n* = 9. **p < 0.01, ***p < 0.001, compared with the control group; ^#^*p* < 0.05, ^##^*p* < 0.01, ^###^*p* < 0.001, compared with the model group. ZZD: Zhizhu Decoction; L-ZZD: low-dose ZZD treatment group; M-ZZD: medium-dose ZZD treatment group; H-ZZD: high-dose ZZD treatment group.

Intestinal neurotransmitters are mainly distributed in the submucosal nerve plexus and intermuscular nerve plexus of the intestinal tract, regulating intestinal secretion and movement, and playing an important role in the normal function of the intestinal tract. In this study, the neurotransmitters Ach, SP, 5-HT, and VIP in the colon were analyzed by ELISA (Figure 3), and it was found that compared with the control group, the expressions of Ach, SP and 5-HT were significantly decreased in the STC model group, while the expression of VIP was significantly increased (*p* < 0.05). And medium-dose and high-dose of ZZD significantly reversed the decreased expression of Ach, SP, and 5-HT and the increased expression of VIP in the model group in a dose-dependent manner (*p* < 0.05), playing a neurotransmitter regulating role, which may have a positive effect on the function of the enteric nervous system (ENS).

### LC-MS targeting neurotransmitter analysis in STC mice

**Figure 4.**
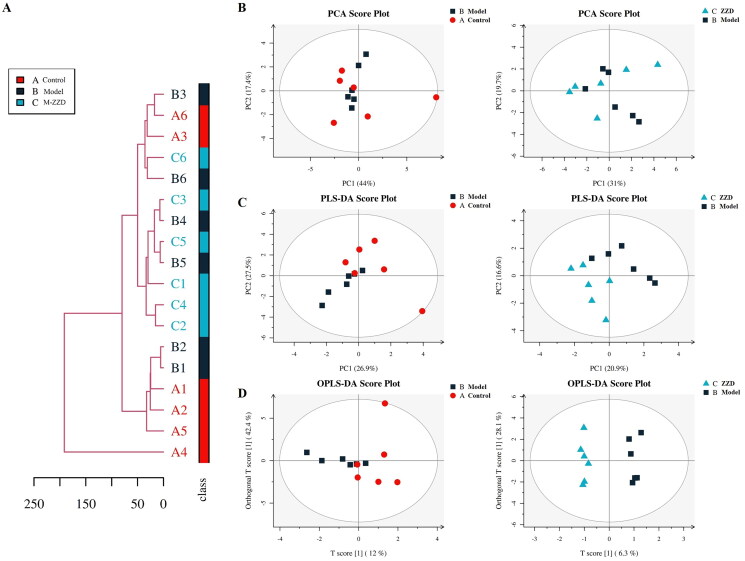
Overall tree diagram and multivariate statistical analysis of differences in neurotransmitters among the group. (A) The overall tree diagram representing the similarity between samples is formed by calculating the distance matrix and clustering all samples by hierarchical clustering. (B) Principal Component Analysis (PCA) score plot of model group and control group, ZZD group and model group. (C) Partial Least Squares-Discriminant Analysis (PLS-DA) score plot of model group and control group, ZZD group and model group in the multivariate statistical analysis. (D) Orthogonal Partial Least Squares Discriminant Analysis (OPLS-DA) score plot of model group and control group, ZZD group and model group. *n* = 6. M-ZZD: medium-dose ZZD treatment group.

As the mice treated with a medium dose of ZZD could significantly improve intestinal peristalsis, intestinal tissue injury and regulate the expression of intestinal neurotransmitters, the subsequent experiments were carried out in the medium-dose of ZZD group (M-ZZD). To further clarify the effects of ZZD on intestinal neurotransmitters, LC-MS targeting neurotransmitters was performed in the serum of mice. Firstly, the distance matrix of relevant data of all samples was calculated, and hierarchical clustering (average-linkage) was used to cluster all samples to form a tree diagram of similarity among samples. As shown in Figure 4(A), there were certain clustering differences among each group of samples, indicating differences in the composition of neurotransmitters in each group of mice. In addition, since the metabolome data are multidimensional and highly correlated with some variables, the stoichiometry principle and diversified statistical analysis were applied to reduce and classify the collected multidimensional data. The results of Principal Component Analysis (PCA) and Partial Least Squares-Discriminant Analysis (PLS-DA) were shown in Figure 4(B,C), respectively. The samples in the control group and the model group were discrete under PC1 and PC2 dimensions, while the discrete distribution between the model group and the ZZD group was more obvious, indicating that there were significant differences between the composition of neurotransmitters in the control group and the model/ZZD group. Similarly, the results of Orthogonal Partial Least Squares Discriminant Analysis (OPLS-DA) in the multivariate statistical analysis also showed significant differences in neurotransmitter composition between the control group and the model/ZZD group (Figure 4(D)). Serum metabolomics results further suggested that ZZD might play a role in regulating neurotransmitters in STC mice.

### Effects of ZZD on the AHR signaling pathway in STC mice

**Figure 5.**
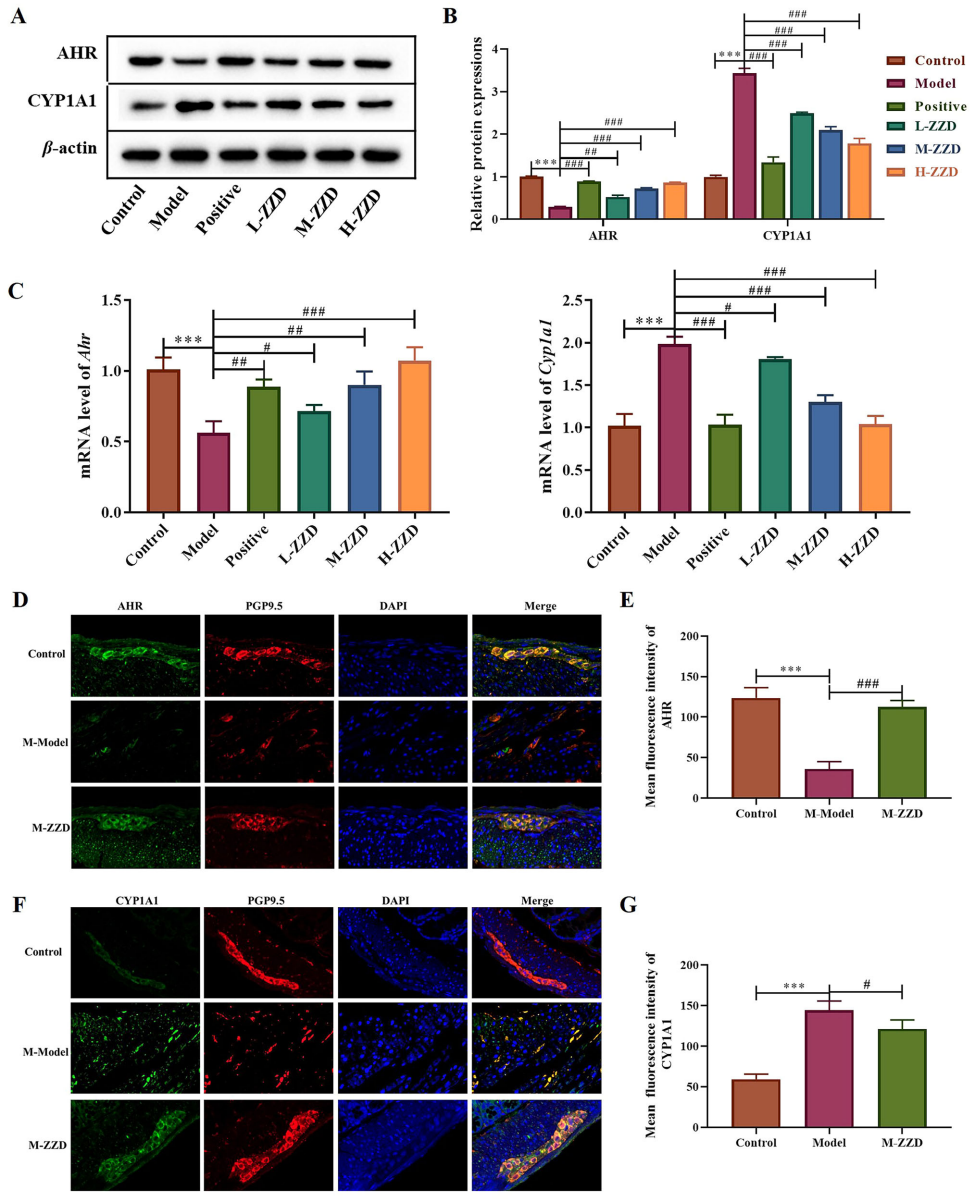
Effects of ZZD on AHR signaling pathway in the colon of STC mice. (A) The protein expression of AHR and CYP1A1 in the colon of STC mice. (B) Densitometry analysis of the intensity of the proteins. (C) The mRNA level of *Ahr* and *Cyp1a1* in the colon of STC mice was detected by qPCR, and the relative expression was normalized to *Actin* expression. (D) Representative images showing immunofluorescence staining of AHR (green) and PGP9.5 (red). Magnification, ×400. (E) Mean fluorescence intensity of AHR in the colon. (F) Representative images showing immunofluorescence staining of CYP1A1 (green) and PGP9.5 (red). Magnification, 400×. Nuclei were stained with 4′,6-diamidino-2-phenylindole (blue) in (D,F). (G) Mean fluorescence intensity of CYP1A1 in the colon. Data were shown as mean ± *SD*, *n* = 9. ***p < 0.001, compared with the control group; ^#^*p* < 0.05, ^##^*p* < 0.01, ^###^*p* < 0.001, compared with the model group. M-ZZD: medium-dose ZZD treatment group.

AHR is a biosensor of the intestinal neural network, which has been proven to be involved in the signal transduction of intestinal neurons. To investigate the possible mechanism of ZZD regulating STC neurotransmitters, the AHR signaling pathway was analyzed. Western blot results showed that compared with the control group, the expression level of AHR in the colon of STC mice was significantly decreased, while the expression level of CYP1A1 was significantly increased. ZZD reversed the decrease of AHR and the increase of CYP1A1 compared with the STC model group, with statistical significance (*p* < 0.05, Figure 5(A,B)). The mRNA results were consistent with the protein results, ZZD significantly reversed the decrease of *Ahr* gene expression and the increase of *Cyp1a1* gene expression (*p* < 0.05, Figure 5(C)). In addition, immunofluorescence staining of the AHR and CYP1A1 in the colon showed significantly decreased expression of AHR and increased expression of CYP1A1 in the STC model group, which was significantly reversed by ZZD (*p* < 0.05, Figure 5(D–G)). These results suggested that ZZD can regulate the AHR signaling pathway.

### Effects of ZZD on the gut microbiota of STC mice

**Figure 6.**
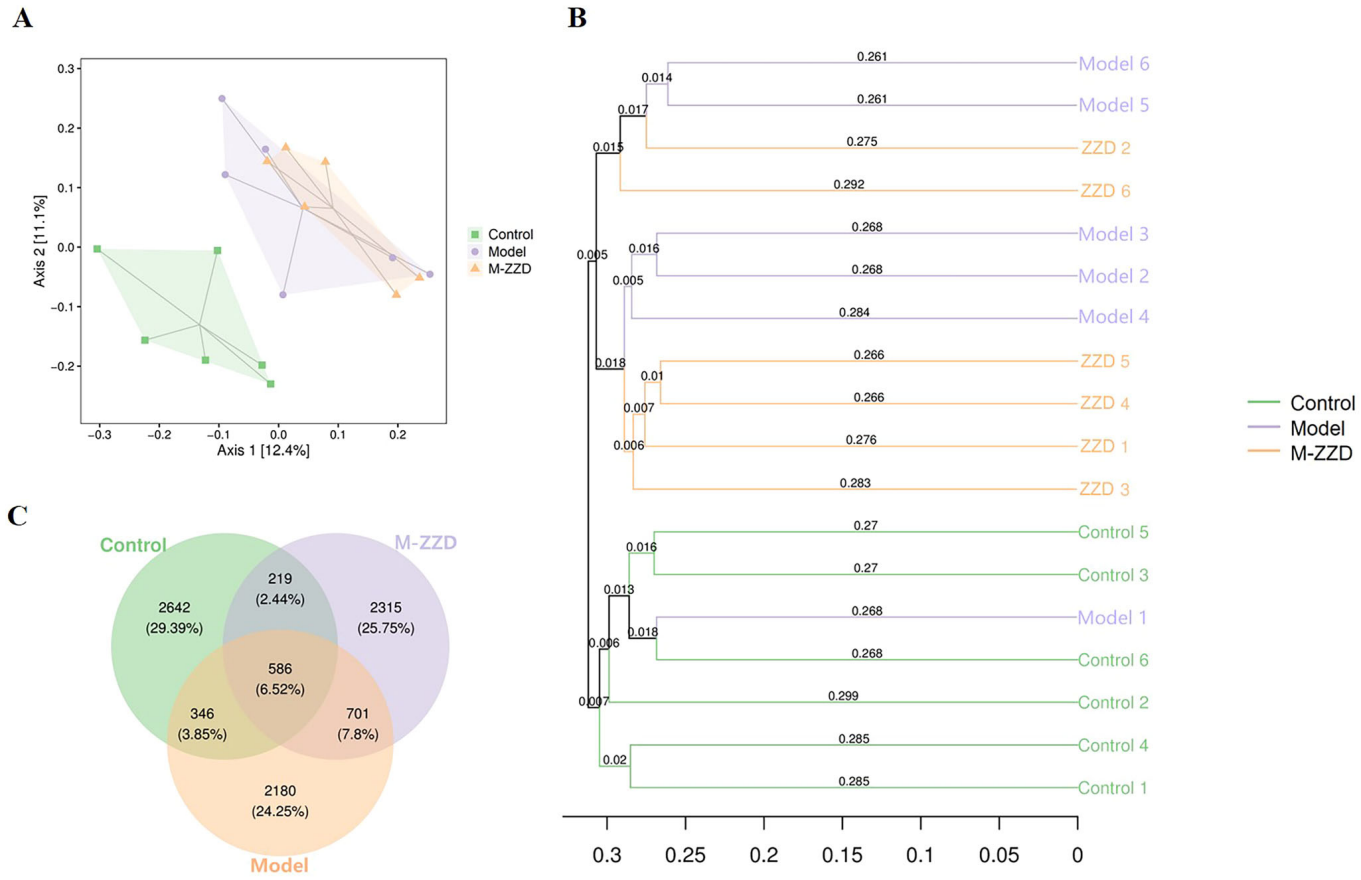
Diversity and composition of fecal microbiota among groups. (A) Two-dimensional ordering diagram of unweighted UniFrac based PCoA. (B) Unweighted UniFrac based UPGMA clustering tree. (C) OTU Venn diagram. *n* = 6. M-ZZD: medium-dose ZZD treatment group.

**Figure 7.**
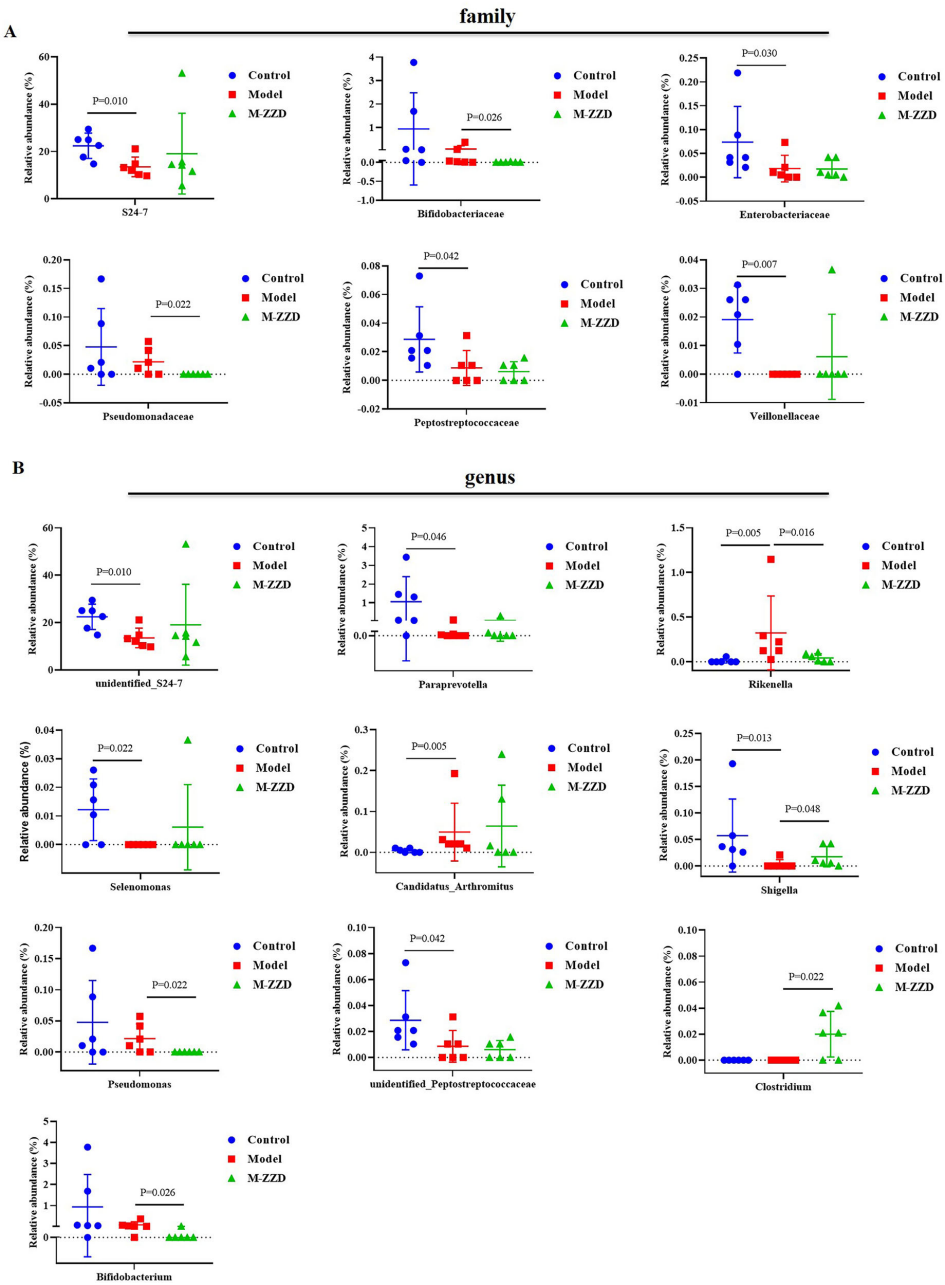
The relative abundance of microflora in each group at the family or genus level. (A) The relative abundance at the family level. (B) The relative abundance at the genus level. Data were shown as mean ± *SD*, *n* = 6. M-ZZD: medium-dose ZZD treatment group.

AHR has been recognized as a regulator of host-microbiome symbiosis. To further elucidate the mechanism of ZZD in the treatment of STC, the gut microbiota of mice feces was analyzed by 16S RNA sequencing. PcoA analysis results showed that the sample distance between the control group and the model/ZZD group was significantly discrete, indicating that the composition and structure of the gut microbiota in the control group were significantly different from those in the model/ZZD group (Figure 6(A)). UPGMA Clustering analysis focuses on describing the similarity clustering among samples, according to the unweighted UniFrac based Clustering tree hierarchical branch length, it can be seen that there were significant clustering differences between the control group and the model group, as well as some significant clustering differences between the model group and the ZZD group, indicating that ZZD had a certain effect on changing the structure of gut microbiota of STC mice (Figure 6(B)). Venn diagram was used for community analysis, and it was found that the unique gut microbiotas in the control group, model group, and ZZD group were 29.39, 24.25, and 25.75%, respectively, indicating that the composition of the gut microbiotas changed after the modeling of STC, and the same changes occurred after ZZD treatment, and their regulation of the gut microbiota was different (Figure 6(C)). The differences in the gut microbiota in each group were further analyzed, and the composition diagram of each sample microflora at family and genus levels were analyzed. As shown in Figure 7, at the family level, compared with the control group, the relative abundances of S24-7, Enterobacteriaceae, Peptostreptococcaceae, and Veillonellaceae in the model group were significantly decreased, and the relative abundances of Bifidobacteriaceae and Pseudomonadaceae in the ZZD group were significantly decreased compared with the model group (*p* < 0.05, Figure 7(A)). In addition, at the genus level, compared with the control group, the relative abundances of *unidentified_S24-7*, *Paraprevotella*, *Selenomonas*, *Shigella*, and unidentified_Peptostreptococcaceae decreased significantly, while the relative abundances of *Rikenella* and *Candidatus_arthromitus* increased significantly in the model group. Compared with the model group, the relative abundances of *Rikenella*, *Pseudomonas*, and *Bifidobacterium* in the ZZD group were significantly decreased, while that of *Shigella* and *Clostridium* were significantly increased (*p* < 0.05, Figure 7(B)). The above results indicated that STC could change the abundance of some microflora, while ZZD could regulate and restore some common differential microflora (such as *Rikenella* and *Shigella* in genus level), and also change the abundance of some unique microflora in the feces of STC mice (such as Bifidobacteriaceae and Pseudomonadaceae in family level, *Pseudomonas*, *Clostridium*, and *Bifidobacterium* in genus level).

**Figure 8.**
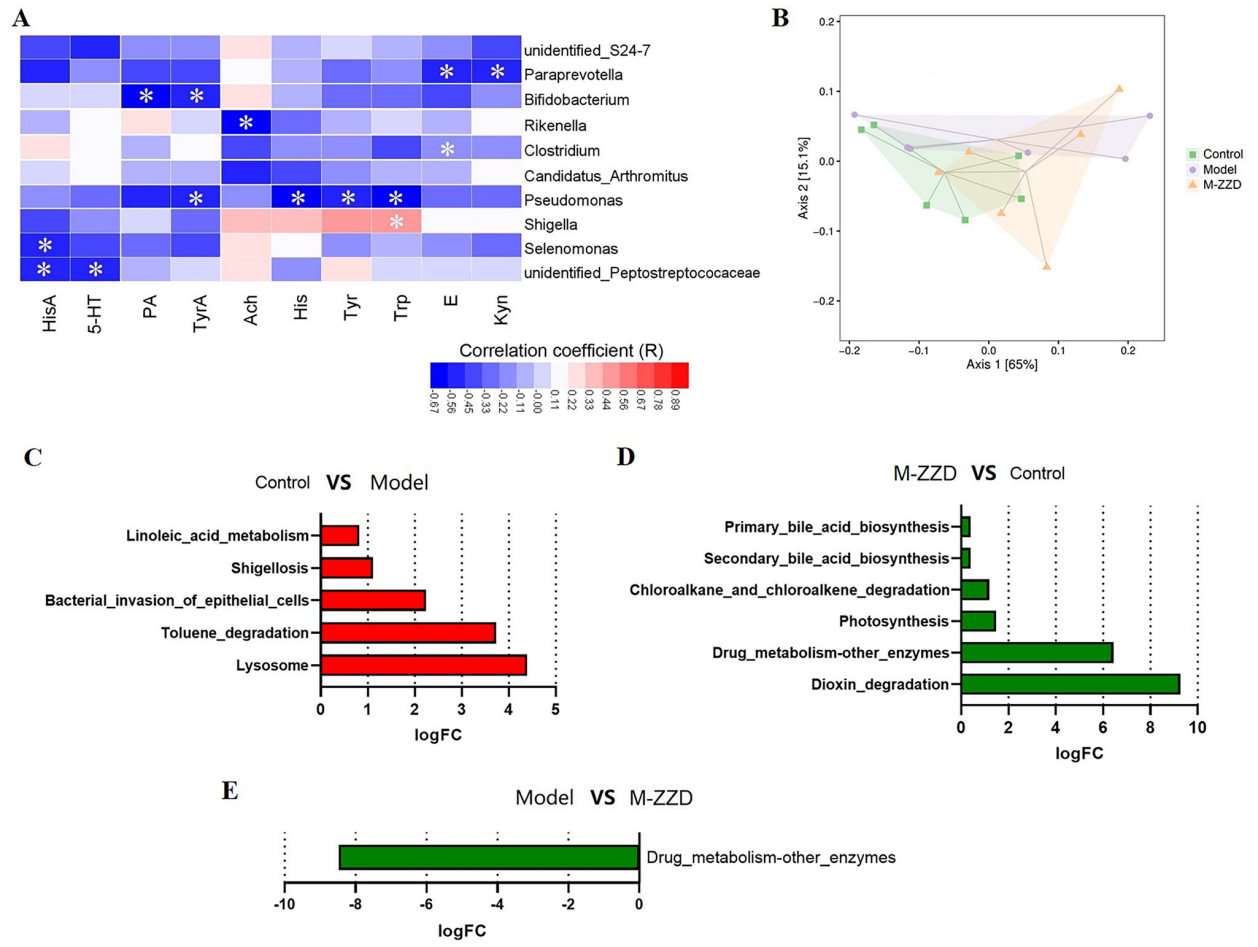
Functional analysis of gut microbiota. (A) Heatmap of correlation analysis between differentially expressed gut microbiota and serum metabolomics. (B)Two-dimensional ordering diagram of Bray-Curtis based PCoA for KEGG orthology (KO). (C) Differences in KEGG metabolic pathways between control and model group. (D) Differences in KEGG metabolic pathways between ZZD and control group. (E) Differences in KEGG metabolic pathways between ZZD and model group. KEGG: Kyoto Encyclopedia of Genes and Genomes. In C-E, the positive value of logFC [log2 (fold change)] on the horizontal axis represents up-regulation, the complex value represents down-regulation, and the ordinate represents different KEGG metabolic pathway labels. M-ZZD: medium-dose ZZD treatment group.

In addition, we analyzed the correlation between the differentially expressed gut microbiota shown in Figure 7(B) and the neurotransmitters in serum metabolomics to elucidate the relationship between ZZD regulation of gut microbiota and neurotransmitter. As shown in Figure 8(A), among the microflora significantly regulated by ZZD, *Rikenella* was significantly negatively correlated with Ach, *Shigella* was significantly positively correlated with Trp (l-tryptophan, CAS: 73-22-3), and *Pseudomonas* was significantly negatively correlated with TyrA (tryptamine, CAS: 61-54-1), His (l-histidine, CAS: 71-00-1), Tyr (l-tyrosine, CAS: 60-18-4) and Trp, and *Bifidobacterium* was negatively significantly correlated with PA (picolinic acid, CAS: 98-98-6) and TyrA (Figure 8(A)). These results suggest that the regulatory effect of ZZD on gut microbiota may be closely related to the changes in the expression of neurotransmitters. To further explore the functional unit differences of gut microbiota among groups, the sample difference Bray-Curtis distance matrix and principal coordinate analysis were used to expand the sample functional differences in low dimensions. The results showed that the sample range among groups only partially overlapped, and there were large non-overlapped areas, suggesting that there were certain differences in the functional units of KEGG orthology among groups (Figure 8(B)). In addition, differential enrichment analysis of the KEGG metabolic pathway (Figure 8(C–E)) showed significant differences in dioxin degradation and drug metabolization-other enzymes between the control group and ZZD group (*p* < 0.05, Figure 8(D)), and significant differences in drug metabolization-other enzymes between the model group and ZZD group (*p* < 0.01, Figure 8(E)). The results indicated that ZZD had the most significant regulation effect on the drug metabolism-other enzyme pathway.

## Discussion

STC is a disease characterized by delayed colon transit, 24 h defecation volume, fecal water content, and ITR can be used as its representative diagnostic indicators (Jani and Marsicano 2018). Studies have shown that compound diphenoxylate is a common method for modeling constipation (Deng et al. 2021). In the present study, it revealed that after continuous intragastric administration of compound diphenoxylate, the defecation volume, fecal water content, and ITR of mice were significantly decreased than those of the control group, and there was a certain degree of pathological damage to the colon, indicating the successful establishment of STC model. As a common Chinese medicine compound, ZZD is beneficial to functional dyspepsia, and this study found that ZZD significantly improved intestinal motility and alleviated colon injury in the STC mouse model. In addition, ZZD significantly increased the expressions of Ach, SP, 5-HT, and decreased the expression of VIP in the colon of STC mice, activated the AHR signaling pathway, and changed the composition of the gut microbiota. Therefore, it could be considered that alleviation of intestinal motility injury in STC mice by ZZD may be associated with the activation of the AHR signaling pathway by gut microbiota to regulate intestinal neurotransmitters.

ZZD is a classic prescription for the treatment of functional gastrointestinal diseases by combining Zhishi and Baizhu, which was first recorded 1700 years ago in a medical classic ‘*Essentials from the Golden Cabinet (Jin Gui Yao Lue)*.’ Zhishi has been proven to exist as a good activity for regulating gastrointestinal motility (Tan et al. 2017), and Baizhu also has an effective regulation effect on the gastrointestinal tract (Chen et al. 2016). ZZD combined with Zhishi and Baizhu can promote gastrointestinal motility and have a significant effect on functional dyspepsia (Wang et al. 2018). The effects of ZZD on gastrointestinal peristaltic activity have been demonstrated in animal experiments (Huang et al. 2012; Chen et al. 2016), and a randomized controlled trial has also confirmed the effectiveness of ZZD in the treatment of functional dyspepsia (Wu et al. 2011). In this study, the components of ZZD were analyzed by HPLC, and naringin was found to be the highest content in ZZD. Studies have shown that naringin has a strong prokinetic activity in gastrointestinal motility dysfunction rats (Jang et al. 2013). The present study found that ZZD could significantly increase the defecation volume, fecal water content, and ITR of STC mice, and significantly alleviate the pathological damage of the colon, which supported the potential therapeutic effect of ZZD on STC. However, the lack of a clear understanding mechanism of ZZD on STC limits its wide application.

The ENS is an important part of the digestive tract (Furness 2012). The excitatory and inhibitory nerves jointly constitute the enteric neural network, which is involved in the regulation of intestinal physiology, including peristalsis (Spencer and Hu 2020). Ach is the most important excitatory neurotransmitter in ENS, which can stimulate gastrointestinal smooth muscle contraction and gland secretion, and promote intestinal peristalsis (Biancani et al. 1988). SP is an excitatory neurotransmitter of the tachykinin family, which can activate Ach and induce smooth muscle contraction, acting as a regulator of Ach (Turner et al. 2007). Studies have shown that there is a significant decrease in the synthesis and release of Ach and SP neurons in the colon tissues of STC patients compared to normal controls (Wattchow et al. 2008; Yik et al. 2011). 5-HT, an important neurotransmitter and paracrine signaling molecule, participates in the regulation of gastric motility through the gastrointestinal smooth muscle contraction mediated by a variety of 5-HT receptors (Gershon and Tack 2007). In addition, VIP is an inhibitory neurotransmitter, which has the function of dilating gastrointestinal sphincter, and the increase of VIP can inhibit gastrointestinal movement (Iwasaki et al. 2019). The present study found that ZZD significantly increased the expressions of Ach, SP, and 5-HT, and decrease the expression of VIP in the colon of STC mice. Serum metabolomics further confirmed that the regulation of ZZD on gastrointestinal function in STC mice may be exerted through regulating neurotransmitters in ENS.

Transcriptional factor AHR is a biosensor of ENS, which is involved in maintaining the excitability of intestinal neurons and regulating intestinal physiology functions (Cervantes-Barragan and Colonna 2018; Obata et al. 2020). Studies have shown that AHR in the intestinal epithelium helps to maintain healthy homeostasis of the intestinal environment, thereby preventing the production of pathogenic changes (Chinen et al. 2015). AHR is a ligand-dependent transcription factor, which can perceive a wide range of exogenous and endogenous molecules (Stockinger et al. 2014). The ligand of AHR can be processed and inactivated by cytochrome P450 (CYP450) family proteins (such as CYP1A1, etc.), and this process is a feedback regulation of the AHR signal (Schiering et al. 2017). The present study found that the protein and mRNA expression of AHR was significantly increased, and the expression of CYP1A1 was significantly decreased after ZZD treatment on STC, suggesting that the AHR signaling pathway was involved in the regulation effects of ZZD on intestinal neurotransmitters of STC.

In addition, AHR was inductively expressed by sensing the microbial environment in the intestinal cavity (Rothhammer and Quintana 2019). Changes in gut microbiota can reduce the excitability of intestinal neurons, leading to intestinal motility disorders and reduced peristalsis (Dimidi et al. 2017). Driven by the natural and social environment, a complex bidirectional network of diet, brain, gut, microbiota, and substances derived from the gut or microbiota is known as the brain-gut-microbiota axis (Dinan and Cryan 2017). On the one hand, the host brain regulates gut function through nerve conduction, which in turn affects the growth of gut microbiota and vice versa. On the other hand, the food residue produced by digestion is fermented by intestinal flora to produce some metabolites. In turn, these biologically active metabolites can affect the microbiota and host gut, and further send signals to the brain. If this axis is disturbed at any level, it will disturb the homeostasis of the body, which may lead to the occurrence and development of diseases (Ganci et al. 2019). Gut microbiota and metabolites act as transit stations in this axis, and currently, increasing evidence from suggests a strong association between gut microbiota and functional constipation (Wang and Yao 2021; Zhang et al. 2021). Microbial treatment can improve the clinical symptoms of STC with fewer adverse reactions (Tian et al. 2016). Secondly, dysbiosis in constipation patients has been reported in several studies that investigated the microbiota in fecal or mucosal samples (Khalif et al. 2005). In addition, germ-free mice developed constipation after receiving fecal microbiota from constipated patients (Ge et al. 2017). In this study, the changes of gut microbiota of mice were analyzed using 16S sequencing, and the results showed that the composition of gut microbiota in the STC group was significantly different from that in the control group, and ZZD also significantly altered the gut microbiota of mice. Correlation analysis between serum metabolomics and differentially expressed gut microbiota showed that the microbiota significantly regulated by ZZD was significantly correlated with several neurotransmitters, including 5-HT, Ach, Tyr, and TyrA. ELISA analysis of colon tissue showed that ZZD could significantly regulate the expression of 5-HT and Ach, suggesting a correlation between the regulatory effects of ZZD on gut microbiota and neurotransmitters. Tryptophan metabolism is an endogenous ligand of AHR, which can bind and activate AHR (Sun et al. 2020). Tyr and TyrA were significantly correlated with differentially expressed gut microbiota regulated by ZZD, further confirming that ZZD may regulate AHR pathway through gut microbiota. In addition, the prediction results of the KEGG pathway showed that the drug metabolism-other enzymes pathway was most significantly different in the ZZD group compared with the control/model group, suggesting that the regulatory effect of ZZD was related to drug metabolism enzymes. More than 55% of clinical drug interactions are caused by changes in the activity of CYP450 enzyme (Anzenbacher and Anzenbacherová 2001), and the members of CYP1, CYP2, and CYP3 families are the most important drug metabolism enzymes, responsible for 70–80% of CYP450-mediated drug metabolism (Ingelman-Sundberg 2004; Guengerich 2006). The induction of CYP450 family proteins involves the classical activation cascade of AHR (Delescluse et al. 2000). Therefore, the results of 16S sequencing further support that ZZD could alleviate STC by regulating AHR signaling pathway through the gut microbiota.

## Conclusions

The present study found that ZZD reversed intestinal peristalsis dysfunction in an STC mouse model, which was manifested as increased 24 h defecation weight, fecal moisture, and ITR, and relieved pathological damage of the colon. In addition, ZZD could regulate the expression of intestinal neurotransmitters, activate the AHR signaling pathway, and alter the composition of gut microbiota in STC mice. The regulation effects of ZZD on intestinal peristalsis in STC mice may be realized by regulating gut microbiota to activate the AHR signaling pathway and promote neuronal excitability. The results of this study can provide experimental basis for the clinical application of ZZD in the treatment of STC.

## Data Availability

The datasets presented in this study can be found in online repositories. The names of the repository/repositories and accession number(s) can be found below: https://www.ncbi.nlm.nih.gov/bioproject/PRJNA778553/.
