# Correction

**DOI:** 10.1080/13880209.2023.2254065

**Published:** 2023-09-04

**Article title:** Zhizhu decoction alleviates slow transit constipation by regulating aryl hydrocarbon receptor through gut microbiota

**Authors:** Wen, Y., Zhan, Y., Tang, S., Liu, F., Wu, R., Kong, P., Li, Q., & Tang, X.

**Journal:** *Pharmaceutical Biology*

**Bibliometrics:** Volume 61, Number 1, pages 111–124

**DOI:** https://doi.org/10.1080/13880209.2022.2157020

This paper contains an error when the article was first published online. The drug dosage in the "Animal treatment" section of the “Materials and methods” section in the article was wrong and did not match the abstract part.

Now, the drug dosage in following sentence “The positive control group was intragastrically given 3 mg/kg mosapride daily, and the L-ZZD, M-ZZD, and H-ZZD group were given 10, 15, and 20 g/kg ZZD, respectively, based on the human-animal drug dose conversion table." under the "Animal treatment" section of the “Materials and methods” section has been corrected as follows "The positive control group was intragastrically given 1.95 mg/kg mosapride daily, and the L-ZZD, M-ZZD, and H-ZZD group were given 5, 10, and 20 g/kg ZZD, respectively, based on the human-animal drug dose conversion table.” and the article has been republished online.

